# Association between the number of pregnancies and cardiac target organ damages: a cross-sectional analysis of data from the Korean women’s chest pain registry (KoROSE)

**DOI:** 10.1186/s12905-023-02514-w

**Published:** 2023-07-17

**Authors:** Hack-Lyoung Kim, Hyun-Jin Kim, Mina Kim, Sang Min Park, Hyun Ju Yoon, Young Sup Byun, Seong-Mi Park, Mi-Seung Shin, Kyung-Soon Hong, Myung-A Kim

**Affiliations:** 1 Division of Cardiology, Department of Internal Medicine, Seoul National University College of Medicine, Seoul National University Boramae Medical Center, Seoul, 07061 Republic of Korea; 2 Division of Cardiology, Department of Internal Medicine, Hanyang University Guri Hospital, Guri, Republic of Korea; 3 Division of Cardiology, Department of Internal Medicine, Korea University Anam Hospital, Seoul, Republic of Korea; 4 Division of Cardiology, Department of Internal Medicine, Eulji University School of Medicine, Nowon Eulji Medical Center, Seoul, Republic of Korea; 5 Division of Cardiology, Department of Internal Medicine, Chonnam National University Hospital, Gwangju, Republic of Korea; 6 Division of Cardiology, Department of Internal Medicine, Inje University Sanggye Paik Hospital, Seoul, Republic of Korea; 7 Division of Cardiology, Department of Internal Medicine, Gil Medical Center, Gachon University College of Medicine, Gil Medical Center, Gyeonggi-do, Republic of Korea; 8 Division of Cardiology, Department of Internal Medicine, Hallym University Medical Center, Chuncheon, Republic of Korea

**Keywords:** Coronary angiography, Diastolic function, Left ventricular mass, Parity, Pregnancy, Target organ damage

## Abstract

**Background:**

Pregnancy increases long-term cardiovascular risk after childbirth, but the mechanisms are unclear. This study was performed to investigate the association between the number of pregnancies and several cardiac target organ damage (TOD) in middle-aged and elderly women.

**Methods:**

Using the database of the nation-wide registry, a total of 1,137 women (mean age 63.0 ± 10.9 years) with stable chest pain undergoing invasive coronary angiography (CAG) were analyzed. Information on the number of pregnancies was obtained through a questionnaire. Obstructive coronary artery disease (CAD), left ventricular (LV) mass index (LVMI) and LV septal annular (e′) velocity were assessed as indicators of cardiac TOD.

**Results:**

Women with higher number of pregnancies (≥ 3) were older (66.3 ± 9.6 vs. 57.4 ± 10.7 years; *P* < 0.001), had more cardiovascular risk factors, and took more cardiovascular medications than those with lower number of pregnancies (< 3). In multivariable analyses, higher number of pregnancies (≥ 3) was associated with obstructive CAD (odds ratio [OR], 1.62; 95% confidence interval [CI], 1.21–2.17; *P* = 0.001), a higher LVMI (> 95 g/m^2^) (OR, 1.46; 95% CI, 1.08–1.98; *P* = 0.013) and a lower septal e′ velocity (< 7 cm/s) (OR, 1.55; 95% CI, 1.12–2.14; *P* = 0.007) even after controlling for potential confounders. As the number of pregnancies increased, the prevalence of CAD and LVMI increased, and the septal e’ velocity gradually decreased (*P* < 0.001 for each).

**Conclusions:**

In women with chest pain undergoing invasive CAG, higher number of pregnancies was associated with multiple cardiac TOD. Parity information should be checked when assessing a woman’s cardiovascular risk.

**Supplementary Information:**

The online version contains supplementary material available at 10.1186/s12905-023-02514-w.

## Introduction

Cardiovascular disease (CVD) is the leading cause of morbidity and mortality for both men and women worldwide [1]. As women have a longer lifespan than men, and the prevalence of CVD is increasing more rapidly after menopause [2], the importance of CVD in women is gradually emerging. According to European data, 55% of women and 43% of men die from CVD [3]. The most effective way to lower cardiovascular risk is to detect high-risk individuals early and provide intensified preventive strategy [4]. Until now, traditional risk factors such as hypertension, diabetes mellitus, dyslipidemia, obesity and smoking have mainly been used as data in screening high-risk subjects [5]. However, these traditional risk factors do not fully represent an individual’s cardiovascular risk. In fact, despite having had myocardial infarction, there was a substantial proportion of patients without traditional risk factors [6]. In this regard, it is necessary to pay attention to non-traditional cardiovascular risk factors.

Pregnancy is one of the women-specific cardiovascular risk factors. Pregnancy places a functional and structural burden on the cardiovascular system and increase cardiovascular risk through multiple pathways [7, 8]. Additionally, pregnancy-related complications such as preterm delivery, hypertensive disorders of pregnancy and gestational diabetes mellitus, are another important cardiovascular risk factors [9–11]. There are several epidemiologic studies showing a positive association between the number pregnancies and the incidence of CVD [12–17]. The results of a meta-analysis also support this finding [18].

The presence of cardiac target organ damage (TOD) predicts future cardiovascular events, so their early detection and treatment is clinically important [19]. Using the nation-wide chest pain registry, KoRean wOmen’S chest pain rEgistry (KoROSE), our group has shown the association between parity and specific cardiac TOD, including obstructive coronary artery disease (CAD) [20] and left ventricular (LV) diastolic dysfunction [21]. However, these studies focused on only one cardiac TOD. To date, there are no research results that have addressed the relationship between the number of pregnancies and multiple cardiac TODs in the same patient. Also, as the number of patients enrolled in the KoROSE increased over time, it was necessary to reassess the correlation between parity and cardiac TOD using a larger number of study patients. We hypothesized that increased number of pregnancies is associated with multiple cardiac TOD, which leads to poor cardiovascular outcomes. Using the KoROSE, this study was performed to investigate the associations of the number of pregnancies with cardiac TOD parameters including findings of invasive coronary angiography (CAG), LV mass index (LVMI) and indicators of LV diastolic function of Korean middle-aged and elderly women. We focused these cardiac TOD parameters because they are closely related to cardiovascular prognosis [22–25] and readily available data from the KoROSE.

## Methods

### Study population

For the analysis of this study, we used database of the KoROSE [20, 21]. From March 2011, women who underwent invasive CAG for the evaluation of CAD have been prospectively enrolled in the KoROSE. Patient registration in the KoROSE is still in progress. The KoROSE was constructed to observe the clinical features and prognoses of Korean women with stable chest pain syndrome. Patients with acute coronary syndrome were excluded. Enrolled patients complained of chest pain, but were in a stable condition. Whether to perform invasive CAG was decided by the attending physician based on the characteristics of chest pain, the patient’s cardiovascular risk, and the results of non-invasive imaging tests or exercise stress tests. A total of 2,253 women were enrolled in the KoROSE, and among them, 1,137 women with accurate parity information were analyzed. The study was conducted in accordance with the Declaration of Helsinki Ethical Principles and Good Clinical Practices. Study protocol was reviewed and approved by the institutional review board (IRB) of Boramae Medical Center (Seoul, Republic of Korea). Approval number of the IRB was 06-2011-222. Written informed consent was obtained from each subject before the registry enrollment.

### Data collection

Body mass index was calculated as weight (kg) divided by the square of height (m^2^). Blood pressure was measured on the right upper arm by a trained nurse using an oscillometric device. Hypertension was defined based on previous diagnosis, current use of antihypertensive medications or systolic/diastolic blood pressure ≥ 140/90 mmHg [26]. Diabetes mellitus was defined based on previous diagnosis, current use of antidiabetic medications, glycated hemoglobin ≥ 6.5% or fasting plasma glucose ≥ 126 mg/dL [27]. Dyslipidemia was defined based on previous diagnosis or low-density lipoprotein cholesterol ≥ 160 mg/dL [28]. Obesity was defined as body mass index ≥ 25 kg/m^2^ [29]. After an overnight fast, blood was aspirated from the antecubital vein to obtain blood levels of the following parameters: white blood cell count, hemoglobin, creatinine, total cholesterol, low-density lipoprotein cholesterol, triglycerides, high-density lipoprotein cholesterol, glucose, glycated hemoglobin and C-reactive protein. Glomerular filtration rate was calculated using the Modification of Diet in Renal Disease (MDRD) study equation [30].

### Transthoracic echocardiography

Transthoracic echocardiography was performed according to the current guidelines [31, 32]. LV ejection fraction was obtained using M-mode tracing, 2D-guided linear measurements or biplane disk summation. During the end-diastolic period, internal dimension (LVIDd) as well as septal wall (IVSd) and posterior wall (LVPWd) thickness of the LV was obtained using M-mode in parasternal long- or short-axis views. Relative wall thickness (RWT) was defined as 2×LVPWd/LVIDd. RWT > 0.42 was used as the criterion for concentric remodeling or hypertrophy. LV mass was obtained using the following Cube formula: LV mass=(0.8)(1.04)×[(LVIDd + LVPWd + IVSd)^3^–IVSd^3^] + 0.6. LVMI was calculated as LV mass indexed to the body surface area. LV hypertrophy was defined as LVMI > 95 g/m^2^. In apical four-chamber view, the movement of the LV septal annulus was measured with the tissue Doppler imaging technique to determine e’ velocity. E and A waves of mitral inflow were also measured using a pulse wave Doppler. Septal e’ velocity < 7 cm/s and E/e’ >15 were considered diastolic dysfunction.

### Invasive CAG

Invasive CAG was performed via the radial or femoral artery according to current guidelines [33, 34]. Obstructive CAD was defined as luminal stenosis ≥ 50% of epicardial coronary arteries. CAD extents were classified as 1-, 2- or 3-vessel disease depending on the number of vessels with ≥ 50% stenosis.

### Statistical analysis

Numbers are expressed as mean ± SD or n (%). For univariable comparisons, study patients were stratified into 2 groups according to median value of the number of pregnancies (≥ 3 *vs*. <3). Student’s t-test and chi-square test were used for the comparisons of continuous and categorized variables, respectively, between the two groups. The association between CAD prevalence and the number of pregnancies was estimated with chi-square test of linear by linear association. Comparisons of mean value of LVMI and septal e’ were assessed using one-way analysis of variances (ANOVA). The adjusted risks of higher number of pregnancies (≥ 3) for target organ damages were obtained using multiple binary logistic regression analysis. Three different multivariable analyses were performed with higher number of pregnancy (≥ 3) as common independent variable, and obstructive CAD, LVMI > 95 g/m^2^, and septal e′ velocity < 7 cm/s as dependent variables in each multivariable model. Following clinical covariates were adjusted during the multivariable analysis: age, body mass index, hypertension, diabetes mellitus and dyslipidemia. Major cardiovascular risk factors, including age, diabetes mellitus, hypertension, dyslipidemia, and smoking, were controlled for through 1:1 propensity score matching (nearest neighbor method within a 0.2 caliper size) between patients with a pregnancy number of ≥ 3 and < 3. Multivariable analysis was also performed using the matched dataset to demonstrate an independent association between a higher number of pregnancies (≥ 3) and cardiac TOD. SPSS statistical package version 20 (IBM Corp, Armonk, NY USA) and R (version 4.2.3; R Foundation for Statistical Computing, Vienna, Austria) were used for the statistical analysis. A *P* value of < 0.05 was considered statistically significant.

## Results

**Fig. 1:**
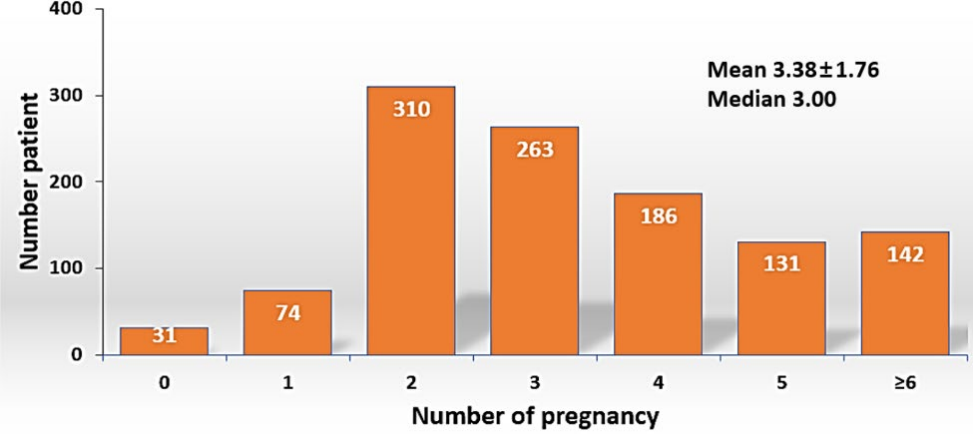
Distribution of number of pregnancies

The mean and median values of the number of pregnancies among the study patients were 3.38 ± 1.76 and 3.00, respectively (Fig. 1). Patients were stratified into two groups according to the number of pregnancies: higher (≥ 3, n = 722) and lower (< 3, n = 415) numbers of pregnancies.

### Clinical characteristics according to the number of pregnancies

The clinical characteristics of the study patients according to the number of pregnancies are shown in Table 1. Patients with higher number of pregnancies were older (66.3 ± 9.6 vs. 57.4 ± 10.7 years, *P* < 0.001) and had more cardiovascular risk factors including hypertension and diabetes mellitus than those with lower number of pregnancies. The laboratory findings did not show any significant differences between the patients with higher and lower pregnancy numbers.

**Table 1:** Clinical characteristics according to pregnancy number

Characteristic	Pregnancy number ≥ 3 (n = 722)	Pregnancy number < 3 (n = 415)	*P*
Age, years	66.3 ± 9.6	57.4 ± 10.7	< 0.001
Weight, kg	59.4 ± 9.1	60.0 ± 9.6	0.245
Height, cm	153 ± 5	155 ± 5	< 0.001
Body mass index, kg/m^2^	25.0 ± 3.6	24.8 ± 3.7	0.240
Systolic blood pressure, mmHg	129 ± 17	128 ± 18	0.552
Diastolic blood pressure, mmHg	76.8 ± 10.9	76.9 ± 11.7	0.977
Heart rate, per minute	74.7 ± 13.4	76.0 ± 14.0	0.247
*Cardiovascular risk factors*			
Hypertension	419 (58.0)	184 (44.3)	< 0.001
Diabetes mellitus	215 (30.0)	99 (24.1)	0.035
Dyslipidemia	163 (22.6)	114 (27.5)	0.064
Current smoking	20 (2.8)	14 (3.4)	0.565
Obesity (body mass index ≥ 25 kg/m^2^)	326 (46.5)	169 (42.6)	0.208
*Major laboratory findings*			
White blood cell count, per *µ*L	6945 ± 2592	6920 ± 3005	0.885
Hemoglobin, g/dL	12.6 ± 1.3	12.8 ± 1.2	0.209
GFR, mL/min/1.73m^2^	85.2 ± 32.8	87.3 ± 28.7	0.315
Total cholesterol, mg/dL	176 ± 44	181 ± 45	0.111
LDL cholesterol, mg/dL	104 ± 36	107 ± 38	0.314
Triglyceride, mg/dL	123 ± 103	119 ± 59	0.470
HDL cholesterol, mg/dL	49.7 ± 13.2	50.9 ± 13.1	0.217
Glucose, mg/dL	126 ± 60	119 ± 61	0.070
Glycated hemoglobin, %	6.34 ± 1.18	6.28 ± 1.20	0.631
C-reactive protein, mg/dL	0.70 ± 2.51	0.70 ± 2.44	0.993

Numbers are expressed as mean ± SD or n (%). GFR, glomerular filtration rate; LDL, low-density lipoprotein; HDL, high-density lipoprotein

### Cardiac TOD parameters according to the number of pregnancies

**Fig. 2:**
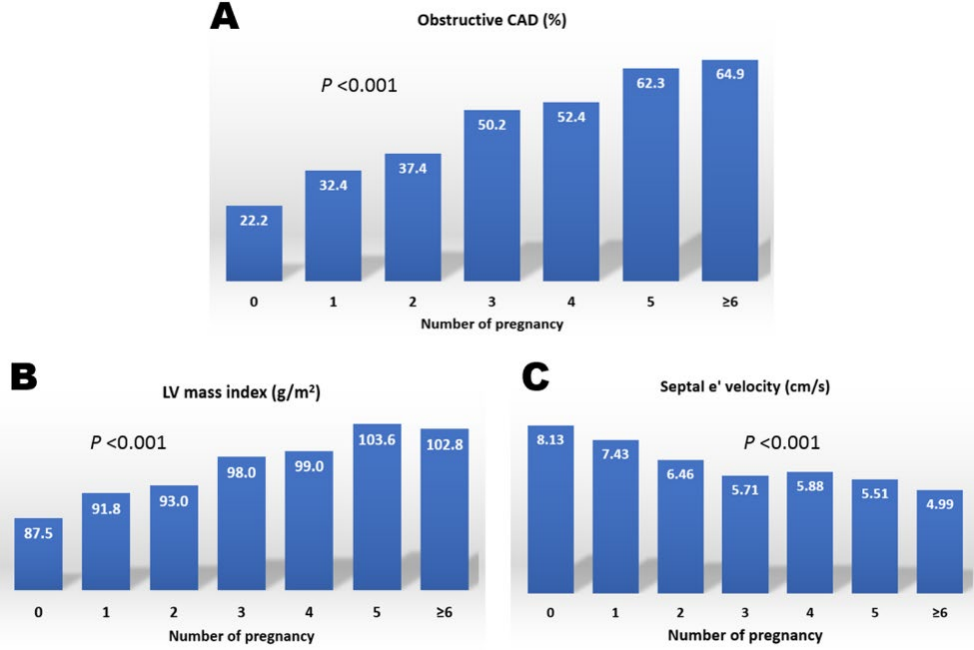
Associations of number of pregnancies with prevalence of obstructive CAD (**A**), LV mass index (**B**) and LV septal e′ velocity (**C**) CAD, coronary artery disease; LV, left ventricular

Comparisons of cardiac TOD parameters between patients with the higher and lower numbers of pregnancies are shown in Table 2. Patients with higher number of pregnancies had lower LV ejection fraction, higher LVMI, lower septal e′ velocity, higher E/e′ and more extensive obstructive CAD, compared to those with lower number of pregnancies (*P* < 0.05 for each). As the number of pregnancies increased, the prevalence rate of obstructive CAD and LVMI increased, and septal e′ velocity decreased proportionally (*P* < 0.001 for each) (Fig. 2).

**Table 2:** Parameters of target organ damage according to pregnancy number

Characteristic	Pregnancy number ≥ 3 (n = 722)	Pregnancy number < 3 (n = 415)	*P*
LV ejection fraction, %	59.4 ± 8.9	60.5 ± 7.9	0.049
LV ejection fraction < 55%	115 (18.0)	43 (12.1)	0.016
RWT	0.39 ± 0.08	0.38 ± 0.07	0.081
RWT > 0.42	175 (28.6)	97 (27.6)	0.739
LV mass index	100.2 ± 30.1	92.4 ± 27.1	< 0.001
LV mass index > 95 g/m^2^	292 (50.9)	121 (35.5)	< 0.001
Septal e’ velocity, cm/s	5.58 ± 1.86	6.73 ± 2.32	< 0.001
Septal e’ velocity < 7 cm/s	448 (74.5)	189 (55.8)	< 0.001
Septal E/e’	11.9 ± 5.2	10.5 ± 4.1	< 0.001
Septal E/e’ >15	87 (17.3)	32 (10.1)	0.004
Obstructive CAD, yes	384 (55.9)	136 (35.4)	< 0.001
*CAD extent*			0.001
Insignificant	303 (44.1)	248 (64.6)	
One-vessel disease	229 (33.3)	90 (23.4)	
Two-vessel disease	103 (15.0)	32 (8.3)	
Three-vessel disease	52 (7.6)	14 (3.6)	

Numbers are expressed as mean ± SD or n (%). LV, left ventricular; RWT, relative wall thickness; LA, left atrial; CAD, coronary artery disease

### Independent association between the number of pregnancies and cardiac TOD

Adjusted risks of higher number of pregnancies (≥ 3) for obstructive CAD, increased LVMI and LV diastolic dysfunction are shown in Table 3. In each separate model, higher number of pregnancies was significantly associated with obstructive CAD (odds ratio [OR], 1.62; 95% confidence interval [CI], 1.21–2.17; *P* = 0.001), LV hypertrophy (LVMI > 95 g/m^2^) (OR, 1.46; 95% CI, 1.08–1.98; *P* = 0.013) and LV diastolic dysfunction (e′ velocity < 7 cm/s) (OR, 1.55; 95% CI, 1.12–2.14; *P* = 0.007) even after controlling for confounding effects of age and other important clinical covariates.

**Table 3:** Adjusted risk of higher number of pregnancy (≥ 3) for obstructive CAD, increased LV mass index and LV diastolic dysfunction

Dependent variable	OR (95% CI)	*P*
Obstructive CAD	1.62 (1.21–2.17)	0.001
LV mass index > 95 g/m^2^	1.46 (1.08–1.98)	0.013
Septal e′ velocity < 7 cm/s	1.55 (1.12–2.14)	0.007

Following variables were adjusted: age, body mass index, hypertension, diabetes mellitus and dyslipidemia. CAD, coronary artery disease; LV, left ventricular; OR, odds ratio; CI, confidence interval

### Cardiac TOD in nulliparity

We analyzed the data by dividing the study patients into two groups: patients with a pregnancy number ≥ 1 (n = 1106) vs. <1 (= nulliparity, n = 31). Nulliparity women were younger and had fewer cardiovascular risk factors (**Supplementary Table **S1). Also, nulliparity women had a lower LVMI, more favorable LV diastolic function, and less obstructive CAD (**Supplementary Table **S2). However, nulliparity was not associated with cardiac TOD in multivariable analysis (**Supplementary Table **S3).

### Propensity score matching analysis

We corrected for differences in major cardiovascular risk factors between patients with more than 3 and less than 3 pregnancies through 1:1 propensity score matching analysis (**Supplementary Table **S4). Multivariable analysis showed that a greater number of pregnancies (≥ 3) were associated with obstructive CAD and LV diastolic dysfunction but not with LVMI. Although LVMI was not statistically significant, there was a numerical trend, which is consistent with the results of unmatched analysis (**Supplementary Table **S5).

## Discussion

### Main findings

The main finding of this study is that higher number of pregnancies (≥ 3) was associated with higher prevalence of obstructive CAD, greater LVMI and lower septal e′ velocity than lower number of pregnancies (< 3) in Korean middle-aged and elderly women undergoing invasive CAG. This associations were significant even after controlling for potential cofounders. To the best of our knowledge, this is the first study showing the association between parity and multiple cardiac TOD in the same patient.

### Previous studies on the association between the number of pregnancies and cardiovascular risk

Many epidemiologic studies have shown that the number of pregnancies is positively associated with cardiovascular risk. Parikh et al. analyzed data of large number of women (n = 1,332,062) from the Swedish population registry, and demonstrated that the number of pregnancies was associated with incident maternal CVD in a J-shaped fashion [12]. In another study of 2,357 women who were followed up for 28 years through the Framingham Heart Study, the incidence rate of coronary heart disease was 1.6 times higher in women with number of pregnancies ≥ 6 compared to women who had never been pregnant [13]. Lawlor et al. also reported that each additional child increased the age-adjusted risk of coronary heart disease by 30% for women with at least 2 children [14]. A study that examined 16,515 women in Sweden showed that women with grand multiparous women (≥ 5 children) had an increased risk of CVD by 1.6-fold compared to those with 2 children [17]. In a meta-analysis of 3,089,929 women from 10 cohort studies, it has been shown that ever parity increased the CVD risk by 14% compared to nulliparity [18].

Few studies have analyzed the mechanisms for which increased number of pregnancies has poor cardiovascular prognosis. Previously, our group has shown the associations of increased number of pregnancies with higher prevalence of obstructive CAD [20] and LV diastolic dysfunction [21] using the KoROSE database. However, those studies analyzed a smaller number of study patients [20] and focused on only one TOD [20, 21]. We showed the association between parity and multiple cardiac TODs, which provides stronger evidence for increased cardiovascular risk in women with higher number of pregnancies.

### Mechanisms

There are several hypotheses that explain the association between higher number of pregnancies and increased maternal cardiovascular risk. It has been reported that a single birth is associated with an average weight gain of 2–3 kg, which increases the risk of overweight or obesity even years after childbirth [35]. Long-term metabolic abnormalities such as dyslipidemia and hyperglycemia due to pregnancy are other problems that increases maternal cardiovascular risk [36, 37]. Also, the incidence of pregnancy complications will increase as the number of pregnancies increases.

Pregnancy associated complications such as gestational hypertension, pre-eclampsia, gestational diabetes, low birth weight, preterm birth and miscarriages are all associated with maternal cardiovascular risk [9–11]. Obstructive CAD, LV mass and LV diastolic function are closely associated with the development of future cardiovascular events [22–25]. There are few data on the relationship between these strong prognostic factors and the number of pregnancies. Our study suggests that obstructive CAD, LV hypertrophy and LV diastolic dysfunction are mediators that explains, at least in part, the association between the number of pregnancies and the occurrence of cardiovascular events.

### Clinical implications

For women, it is important to understand risk factors associated with pregnancy and to incorporate them into practical preventive therapy in order to reduce the cardiovascular risk. However, women-specific risk factors are under recognized [38]. As the number of pregnancies is easily identified with a simple question, obtaining this information should be a routine in cardiovascular examinations in women. Combining the results of previous studies and ours, when the number of pregnancies is three or more, the cardiovascular risk increases compared to women with fewer or no pregnancies. Therefore, it is better to pay more attention and actively examine subclinical TOD for women with higher number of pregnancies. According to the results of this study, although there are various indicators of cardiac TOD, it would be desirable to examine them with interest in CAD, LV hypertrophy, and LV diastolic dysfunction. Of course, the association between other cardiac TOD indicators and the number of pregnancies should be elucidated through additional studies. Better understanding of the association between higher number of pregnancies and cardiac TOD could be explored in additional studies to identify the areas of modifiable risk in women in order to reduce their cardiovascular risk.

Several studies have suggested that the relationship between the number of pregnancies and the risk of cardiovascular disease in women follows a J- or U-shaped curve [12, 39, 40]. These findings indicate that women with one or two pregnancies had a lower cardiovascular risk compared to those who had never been pregnant, suggesting that a few pregnancies may have a protective effect on cardiovascular health. Taken together, it becomes apparent that while having a high number of pregnancies (3 or more) elevates cardiovascular risk, having one or two pregnancies may be neutral or even beneficial for women’s cardiovascular system.

### Study limitations

There are several limitations of this study. First, this cross-sectional study could not determine the causal relationship between the number of pregnancies and cardiac TOD parameters. Secondly, only the confounding effects of several important clinical covariates were adjusted in multivariable analyses. Our results may have been influenced by other variables such as socioeconomic status, lifestyle, environmental factors, and pregnancy associated complications, which are not included in the registry database. Thirdly, while the cardiovascular risk in the nulliparity group may be a significant concern, the impact of nulliparity on TOD could not be effectively analyzed in our study due to the limited sample size. To properly assess this, future research should conduct a comparative analysis with a larger cohort of nulliparous women. Lastly, since our study results were obtained from Koreans who had undergone invasive CAG, caution is required when applying to other groups.

## Conclusions

In Korean women undergoing invasive CAG, increased number of pregnancies was associated with higher prevalence of obstructive CAD, increased LVMI and LV diastolic dysfunction. Efforts should be made to reduce cardiovascular risk by paying more attention to women with a higher number of pregnancies. Also, obtaining a pregnancy history should be an integral part of women’s cardiovascular risk assessment. Further well-designed prospective studies are needed to support our findings and to clarify causal relationships between parity and cardiac TOD.

## Electronic supplementary material

Below is the link to the electronic supplementary material.

**Supplementary Table S1**. Clinical characteristics according to pregnancy number.
**Supplementary Table S2**. Parameters of target organ damage according to pregnancy number.
**Supplementary Table S3**. Adjusted risk of nulliparity for obstructive CAD, increased LV mass index and LV diastolic dysfunction.
**Supplementary Table S4**. Clinical characteristics according to pregnancy number before and after propensity score matching.
**Supplementary Table S5**. The risk of higher number of pregnancy (≥3) for obstructive CAD, increased LV mass index and LV diastolic dysfunction in propensity score matched set.

## Data Availability

The datasets used and/or analysed during the current study available from the corresponding author on reasonable request.

## Abbreviations

ANOVA: analysis of variance
CAD: coronary artery disease
CAG: coronary angiography
CVD: cardiovascular disease
IRB: institutional review board
IVSd: interventricular septal wall thickness during diastole
KoROSE: KoRean wOmen’s chest pain rEgistry
LV: left ventricular
LVIDd: left ventricular internal dimension during diastole
LVMI: left ventricular mass index
MDRD: Modification of Diet in Renal Disease
RWT: relative wall thickness
TOD: target organ damage
