# Supplementary material for: Association between the number of pregnancies and cardiac target organ damages: a cross-sectional analysis of data from the Korean women’s chest pain registry (KoROSE)
Source: BMC Womens Health. 2023 Jul 17;23:377. doi: 10.1186/s12905-023-02514-w (PMC10353181; doi:10.1186/s12905-023-02514-w)
Supplement: Supplementary file 1 — Supplementary Table S1. Clinical characteristics according to pregnancy number. Supplementary Table S2. Parameters of target organ damage according to pregnancy number. Supplementary Table S3. Adjusted risk of nulliparity for obstructive CAD, increased LV mass index and LV diastolic dysfunction. Supplementary Table S4. Clinical characteristics according to pregnancy number before and after propensity score matching. Supplementary Table S5. The risk of higher number of pregnancy (≥3) for obstructive CAD, increased LV mass index and LV diastolic dysfunction in propensity score matched set. [file 12905_2023_2514_MOESM1_ESM.docx]

**Supplementary Table S1. Clinical characteristics according to pregnancy number**

| **Characteristic** | **Pregnancy number <1**  **(n=31)** | **Pregnancy number ≥1**  **(n=1106)** | ***P*** |
| --- | --- | --- | --- |
| Age, years | 45.6±14.6 | 63.5±10.4 | <0.001 |
| Weight, kg | 57.7±12.7 | 59.7±9.1 | 0.242 |
| Height, cm | 158±7 | 154±5 | <0.001 |
| Body mass index, kg/m^2^ | 23.1±5.5 | 25.0±3.5 | 0.072 |
| Systolic blood pressure, mmHg | 129±17 | 128 ± 18 | 0.906 |
| Diastolic blood pressure, mmHg | 75.2±11.9 | 76.9±11.2 | 0.521 |
| Heart rate, per minute | 84.0±19.1 | 75.0±13.4 | 0.102 |
| *Cardiovascular risk factors* |  |  |  |
| Hypertension | 9 (29.0) | 594 (53.7) | 0.007 |
| Diabetes mellitus | 9 (29.0) | 305 (27.8) | 0.883 |
| Dyslipidemia | 7 (22.6) | 270 (24.4) | 0.815 |
| Current smoking | 2 (6.5) | 32 (2.9) | 0.251 |
| Obesity (body mass index ≥25 kg/m^2^) | 8 (26.7) | 487 (45.6) | 0.040 |
| *Major laboratory findings* |  |  |  |
| White blood cell count, per *μ*L | 7928±2610 | 6912±2748 | 0.068 |
| Hemoglobin, g/dL | 12.9±1.0 | 12.7±1.3 | 0.350 |
| GFR, mL/min/1.73m^2^ | 92.9±31.7 | 85.8±31.4 | 0.263 |
| Total cholesterol, mg/dL | 182±57 | 178±44 | 0.693 |
| LDL cholesterol, mg/dL | 108±49 | 105±37 | 0.723 |
| Triglyceride, mg/dL | 112±38 | 122±38 | 0.693 |
| HDL cholesterol, mg/dL | 49.3±13.1 | 50.1±13.2 | 0.786 |
| Glucose, mg/dL | 106±34 | 124±61 | 0.182 |
| Glycated hemoglobin, % | 6.62±1.50 | 6.31±1.19 | 0.609 |
| C-reactive protein, mg/dL | 0.50±1.80 | 0.70±2.50 | 0.686 |

Numbers are expressed as mean±SD or n (%). GFR, glomerular filtration rate; LDL, low-density lipoprotein; HDL, high-density lipoprotein.

**Supplementary Table S2. Parameters of target organ damage according to pregnancy number**

| **Characteristic** | **Pregnancy number <1**  **(n=31)** | **Pregnancy number ≥1**  **(n=1106)** | ***P*** |
| --- | --- | --- | --- |
| LV ejection fraction, % | 58.7±8.5 | 59.9±8.6 | 0.496 |
| LV ejection fraction <55% | 7 (22.5) | 151 (15.6) | 0.120 |
| RWT | 0.37±0.06 | 0.39±0.07 | 0.114 |
| RWT >0.42 | 5 (16.1) | 267 (28.5) | 0.299 |
| LV mass index | 87.4±33.5 | 97.5±29.1 | 0.089 |
| LV mass index >95 g/m^2^ | 6 (19.3) | 407 (45.7) | 0.031 |
| Septal e' velocity, cm/s | 8.12±2.25 | 5.94±2.08 | <0.001 |
| Septal e' velocity <7 cm/s | 6 (19.3) | 631 (68.8) | <0.001 |
| Septal E/e' | 8.77±2.54 | 11.5±4.9 | 0.010 |
| Septal E/e' >15 | 0 | 119 (14.9) | 0.050 |
| Obstructive CAD, yes | 6 (19.3) | 514 (49.2) | 0.006 |
| *CAD extent* |  |  | 0.019 |
| Insignificant | 25 (80.6) | 592 (53.5) |  |
| One-vessel disease | 3 (9.7) | 316 (28.6) |  |
| Two-vessel disease | 3 (9.7) | 132 (11.9) |  |
| Three-vessel disease | 0 | 66 (6.0) |  |

Numbers are expressed as mean±SD or n (%). LV, left ventricular; RWT, relative wall thickness; LA, left atrial; CAD, coronary artery disease.

**Supplementary Table S3. Adjusted risk of nulliparity for obstructive CAD, increased LV mass index and LV diastolic dysfunction**

| **Dependent variable** | **OR (95% CI)** | ***P*** |
| --- | --- | --- |
| Obstructive CAD | 0.55 (0.19-1.59) | 0.552 |
| LV mass index >95 g/m^2^ | 0.70 (0.26-1.90) | 0.492 |
| Septal e′ velocity <7 cm/s | 0.47 (0.14-1.56) | 0.220 |

Following variables were adjusted: age, body mass index, hypertension, diabetes mellitus and dyslipidemia. CAD, coronary artery disease; LV, left ventricular; OR, odds ratio; CI, confidence interval.

**Supplementary Table S4. Clinical characteristics according to pregnancy number before and after propensity score matching**

|  | **Before propensity score matching** | | | **After propensity score matching** | | |
| --- | --- | --- | --- | --- | --- | --- |
| **Variables** | **Pregnancy number <3 (n=415)** | **Pregnancy number ≥3 (n=722)** | ***P*** | **Pregnancy umber <3 (n=331)** | **Pregnancy number ≥3 (n=331)** | ***P*** |
| Age, years | 57.4±10.7 | 66.3±9.6 | <0.001 | 60.3±9.2 | 60.3±9.3 | 0.930 |
| Body mass index, kg/m^2^ | 24.82±3.9 | 25.0±3.5 | 0.243 | 25.0±3.7 | 25.0±3.3 | 0.926 |
| Diabetes mellitus | 99 (23.8) | 215 (29.7) | 0.031 | 85 (25.6) | 83 (25.0) | 0.860 |
| Hypertension | 184 (44.3) | 419 (58.0) | <0.001 | 157 (47.4) | 152 (45.9) | 0.682 |
| Dyslipidemia | 114 (27.4) | 163 (22.5) | 0.064 | 92 (27.7) | 85 (25.6) | 0.534 |
| Current smoking | 14 (3.3) | 20 (2.7) | 0.565 | 10 (3.0) | 11 (3.3) | 0.818 |

Numbers are expressed as mean±SD or n (%).

**Supplementary Table S5. The risk of higher number of pregnancy (≥3) for obstructive CAD, increased LV mass index and LV diastolic dysfunction in propensity score matched set**

| **Dependent variable** | **OR (95% CI)** | ***P*** |
| --- | --- | --- |
| Obstructive CAD | 1.66 (1.16-2.35) | 0.004 |
| LV mass index >95 g/m^2^ | 1.23 (0.93-1.56) | 0.078 |
| Septal e′ velocity <7 cm/s | 1.43 (1.02-1.67) | 0.037 |

CAD, coronary artery disease; LV, left ventricular; OR, odds ratio; CI, confidence interval.
